# An alternative to MINFLUX that enables nanometer resolution in a confocal microscope

**DOI:** 10.1038/s41377-022-00896-4

**Published:** 2022-06-30

**Authors:** Luciano A. Masullo, Alan M. Szalai, Lucía F. Lopez, Mauricio Pilo-Pais, Guillermo P. Acuna, Fernando D. Stefani

**Affiliations:** 1 Centro de Investigaciones en Bionanociencias (CIBION), Consejo Nacional de Investigaciones Científicas y Técnicas (CONICET), Godoy Cruz 2390, C1425FQD Ciudad Autónoma de Buenos Aires, Buenos Aires, Argentina; 2 Departamento de Física, Facultad de Ciencias Exactas y Naturales, Universidad de Buenos Aires, Güiraldes 2620, C1428EHA Ciudad Autónoma de Buenos Aires, Buenos Aires, Argentina; 3 Department of Physics, University of Fribourg, Chemin du Musée 3, Fribourg, CH-1700 Switzerland

**Keywords:** Super-resolution microscopy, Imaging and sensing

## Abstract

Localization of single fluorescent emitters is key for physicochemical and biophysical measurements at the nanoscale and beyond ensemble averaging. Examples include single-molecule tracking and super-resolution imaging by single-molecule localization microscopy. Among the numerous localization methods available, MINFLUX outstands for achieving a ~10-fold improvement in resolution over wide-field camera-based approaches, reaching the molecular scale at moderate photon counts. Widespread application of MINFLUX and related methods has been hindered by the technical complexity of the setups. Here, we present RASTMIN, a single-molecule localization method based on raster scanning a light pattern comprising a minimum of intensity. RASTMIN delivers ~1–2 nm localization precision with usual fluorophores and is easily implementable on a standard confocal microscope with few modifications. We demonstrate the performance of RASTMIN in localization of single molecules and super-resolution imaging of DNA origami structures.

## Abstract (web summary)

Localization of single fluorescent emitters is key for physicochemical and biophysical measurements at the nanoscale. Recently, the method called MINFLUX has achieved a ~10-fold improvement in spatial resolution over previously developed techniques, reaching molecular resolution with moderate photon counts. However, the technical complexity of this technique has hindered its widespread application. The new technique called RASTMIN provides equivalent resolution to MINFLUX while it can be implemented in standard scanning (confocal) microscopes.

## Graphical abstract

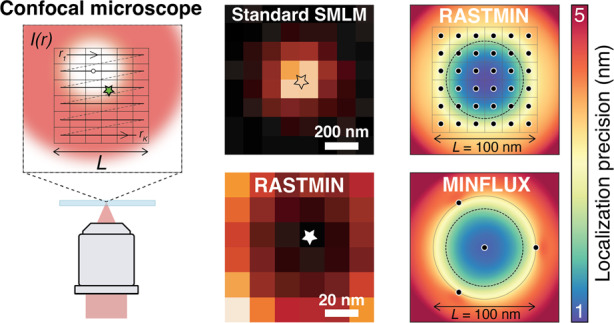

## Introduction

Fluorescence microscopy is a major workhorse in life sciences, biophysics, and physical chemistry, as it allows visualization with great specificity and sensitivity. In particular, the detection of single fluorescent molecules was pioneered more than thirty years ago^1,2^. Since then, single-molecule detection and spectroscopy has evolved into numerous analytical methods capable of providing information beyond ensemble averages with applications in physical chemistry, nanophotonics, and biophysics^3–8^, among other fields. Arguably, the most prominent application is super-resolution imaging by single-molecule localization microscopy^9–11^. Another important application is single-molecule tracking, which reveals molecular trajectories that would be otherwise hidden in the average behavior of an ensemble of unsynchronized molecules^12–16^.

Most commonly, single-molecule detection and tracking are performed in a wide-field configuration using uniform illumination. The molecular positions are determined from a fit to their images recorded with a photodetector array (e.g., an EM-CCD or CMOS camera). Typically, this approach delivers a lateral localization precision in the range of 10 to 50 nm for organic fluorophores under biologically compatible conditions. The achieved localization precision is limited by the photostability of the fluorophores^11,17,18^.

Alternatively, other approaches infer the molecular position from the signal registered upon excitation with a sequence of spatially shifted patterns of light. This type of methods can be interpreted in a common framework^19^ and termed single-molecule localization with sequential structured illumination (SML-SSI). They were first developed for single-molecule tracking using Gaussian beams^20–25^, i.e., the so-called orbital tracking method. More recently, minimal photon fluxes (MINFLUX)^26^ represented a breakthrough by achieving a ~10-fold improvement compared to wide-field camera-based SML, reaching ~1–2 nm localization precision using just a few hundred fluorescence photon counts. So far, MINFLUX remains the most photon-efficient SML method and has been demonstrated in model systems (DNA origami structures)^26–28^, extended to three dimensions in fixed and living cells^29,30^, and it was combined with fluorescence lifetime measurements^28^. Parallelized versions of SML-SSI have also been developed^31–34^ although their higher throughput comes at the expense of a lower resolution. A common feature of SML-SSI methods, and particularly of MINFLUX, is their considerably higher technical complexity when compared to camera-based methods. Possibly for this reason the use of these techniques has been limited to a reduced number of expert groups and its widespread application remains an open challenge.

Here, we introduce RASTMIN (single-molecule localization by RASTer scanning a MINimum of light), a SML-SSI method that delivers equivalent performance compared to MINFLUX and that can be implemented on any scanning fluorescence (e.g. confocal or multiphoton^35,36^) microscope with only minor modifications.

## Results

### Principle of RASTMIN and theoretical localization precision

**Fig. 1:**
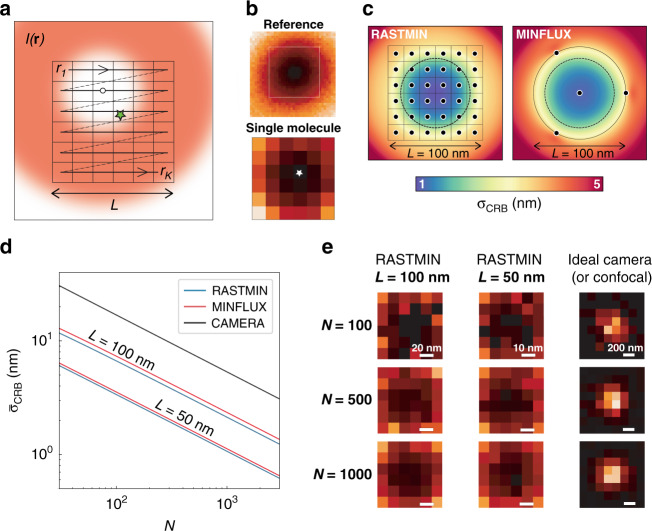
RASTMIN concept. **a** Schematic of the RASTMIN measurement: a beam of light with intensity $I({{{{r}}}})$ that features a minimum is raster-scanned over ${{r}}_{{{i}}} = {{r}}_1, \ldots ,{{r}}_{{{K}}}$ pixels defining a region of size *L* that contains the single emitter (green star). **b** Two images are acquired: a high-SNR, high-resolution image of the excitation beam (Reference) and a low-SNR, low-resolution image formed by the set of photon counts recorded at each pixel *r*_*i*_ (Single molecule). Both images are then used as input to perform a maximum-likelihood estimation of the emitter position. **c** Theoretical localization precision as a function of the emitter position, *σ*_CRB_(x, y), of RASTMIN (*K* = 6 × 6) and MINFLUX for *N* = 1000 and *L* = 100 nm. **d** Average precision $\bar \sigma$_CRB_ of RASTMIN and MINFLUX over the central area (diameter of 0.75*L*) of the scanned region (black dotted circle in **c** as a function of *N* for *L* = 100 nm and *L* = 100 nm For MINFLUX, the triangular pattern with a central exposition used in the original publication^26^ was used (see Supplementary Section 1 for details). For comparison, also the $\bar \sigma$_CRB_ for a camera-based localization without readout noise is displayed. **e** Exemplary simulated data for a RASTMIN localization at *L* = 100 nm and *L* = 50 nm as well as a camera-based localization for different values of *N*. In all cases SBR = 4

Figure 1a schematically shows the concept of a RASTMIN measurement in two dimensions. The sample is illuminated with spatially modulated excitation light $I({{{\boldsymbol{r}}}})$comprising a local minimum (ideally a zero) of intensity. The data acquisition of RASTMIN is identical to registering an image with a scanning microscope. Conceptually, RASTMIN consists of comparing two images of the central minimum of $I({{{\boldsymbol{r}}}})$ (Fig. 1b): a reference image obtained with high resolution and high signal-to-noise ratio (SNR), and an image obtained with the single-molecule whose position is to be determined. The reference image of $I({{{\boldsymbol{r}}}})$ may be obtained through averaging multiple raster-scan images of the light intensity minimum over a single fluorescent molecule or a small emitter (e.g. a fluorescent nanoparticle). The single-molecule image used for localization is obtained by raster scanning a sub-diffraction area of the sample where the target molecule is present. The single-molecule fluorescence signal is registered as photon counts ${{{\mathbf{n}}}} = [n_1,n_2, \ldots ,n_K]$ for each pixel $\left[ {{{{\boldsymbol{r}}}}_1,{{{\boldsymbol{r}}}}_2, \ldots ,{{{\boldsymbol{r}}}}_{{{\boldsymbol{K}}}}} \right]$. Then, the position of the molecule is inferred as the parameter that best matches the relative intensity of the single-molecule image in comparison to the reference image, e.g. using maximum-likelihood estimation^19^.

To evaluate the theoretically achievable localization precision of RASTMIN, we calculated the Cramér-Rao lower bound for the localization uncertainty (*σ*_CRB_). As described previously^19^, for a given $I( {{{\boldsymbol{r}}}})$, $\sigma _{\rm{CRB}}$ depends on the position of the emitter (*x,y*), the total number of photons used for the position estimation ($N = \mathop {\sum }\nolimits_{i = 1}^K n_i$), the signal-to-background ratio (*SBR*), and the size of the raster. We will consider a square raster of side *L* and a doughnut-shaped $I({{{\boldsymbol{r}}}})$^19^ with a full width at half-maximum (FWHM) of 300 nm. Figure 1c shows a 2D map of $\sigma _{\rm{CRB}}\left( {x,y} \right)$ for RASTMIN with *L* = 100 nm, *N* = 1000, *K* = 6 × 6, and SBR = 4. Also in Fig. 1c, the *σ*_CRB_(*x*, *y*) computed for MINFLUX with the same *N* and *SBR*, and an equivalent geometry of the excitation pattern (see Supplementary Section 1 for details) is shown for comparison. In both cases the localization error is minimum at the center of the excitation pattern, where it is about 1 nm, and increases outwards. The localization precision remains below 5 nm over an area that extends beyond the excitation pattern. Remarkably, this area is larger for RASTMIN. Figure 1d shows the average precision $\bar \sigma _{\rm{CRB}}$ over the central area of the excitation pattern (black dotted circle in Fig. 1c) vs *N* for RASTMIN and MINFLUX with *L* = 50 nm and *L* = 100 nm at *SBR* = 4. For comparison, Fig. 1d also shows the average precision for a camera-based localization with the same *SBR*. In all cases, only Poisson noise was considered, which is a good approximation for measurements performed with single-photon counting detectors presenting low dark counts, e.g. avalanche photodiodes. For the cameras, the pure Poisson noise situation represents an ideal best-case scenario. Real-world CMOS or CCD cameras present super-Poissonian noise, and thus deliver lower localization precisions.

RASTMIN delivers a localization precision equivalent to MINFLUX. For both methods, the uncertainty scales as $\sigma _{\rm{CRB}} \propto L$. The improvement in localization precision as *L* decreases is ultimately limited by the achievable *SBR*^26,27^. Conceptually, the dependence of *σ*_CRB_ with *L* can be understood as follows. The minimum of $I({{{\boldsymbol{r}}}})$ is well approximated by a paraboloid. Hence, the single-molecule RASTMIN measurement produces a scanned image of a paraboloid with its minimum corresponding to the position of the molecule. For any given *N* and with an equivalent signal-to-noise ratio (*SNR*), such images remain self-similar as *L* decreases, as shown in the examples of Fig. 1e (left and central panels). Furthermore, if *K* is constant, the uncertainty of the localization in units of pixels is independent of *L*. Thus, reducing the pixel size leads to a proportionally smaller localization uncertainty in length units. This cannot be achieved in a single-molecule image obtained by scanning a maximum of light^37^ or with a camera sensor measurement (Fig. 1e, right panel). In this case, the full-width at half maximum (FWHM) of the image cannot be tuned because it is determined by the diffraction limit of light.

From this point of view, MINFLUX could also be interpreted as a minimal image of the excitation beam $I({{{\boldsymbol{r}}}})$ with unconventionally distributed pixels. Remarkably, there is no fundamental advantage in using fewer pixels. In fact, if the measurement involves only Poisson noise, performing a RASTMIN measurement with more pixels (increasing *K*) provides more information and leads to higher localization precisions (Supplementary Section 2). Supplementary Fig. 2 shows curves of $\bar \sigma _{\rm{CRB}}$ vs *K* for different total photon counts *N*. Also, exemplary simulated images for $K = 9,36,100,256$ are shown. The localization precision improves significantly as *K* increases from 4 up to 36, approximately. From then on, the localization precision improves marginally. In practice, the use of very large *K* might be detrimental due to the limited positioning precision of the scanner. We conclude that *K* = 16–100 is a good range for experimental realizations.

It is relevant to discuss the spatial and temporal resolutions of RASTMIN compared to MINFLUX. First, it is important to note that for this type of methods the localization precision for any given photon budget *N* and *SBR* (i.e. the localization photon efficiency) is independent of the time required for the measurement. The localization photon efficiency depends only on the geometry of the illumination beam and the positions of the exposures. On the other hand, the number of exposures *K* is not a parameter that will affect the temporal resolution. The key parameter is the speed of the scanning, i.e., the time required to displace the excitation beam over a given distance in the sample, independently of how many measurements (pixels) are made over that distance.

From an experimental point of view, performing MINFLUX using electro-optical deflectors (EOD)^26,38^ or pulsed-interleaved excitation^28^ provides fast scanning speeds that allow completing an excitation sequence in ~100 μs or ~25 ns, respectively. If the photon detection count rate of the fluorophore is not a limitation, such fast scanning speeds are advantageous for single-molecule tracking measurements. In contrast, for imaging applications, the time needed to obtain a super-resolved image is not determined by the scanning speed but by the sequential localization of all target molecules in the region of interest, which in turn depends on the characteristic times of the ON/OFF cycles of the dyes. The only requirement for the scanning speed is to be faster than the ON-times of the blinking fluorophores, which usually lie between 10 and 500 ms. RASTMIN implemented in a confocal microscope with standard galvanometric scanners can complete an excitation sequence in ~1–20 ms, depending on the particular optical scanner. The typical acquisition time for a super-resolved image is of several minutes for all SMLM techniques, including PALM/STORM, MINFLUX, or RASTMIN.

### Experimental implementation and localization precision

**Fig. 2:**
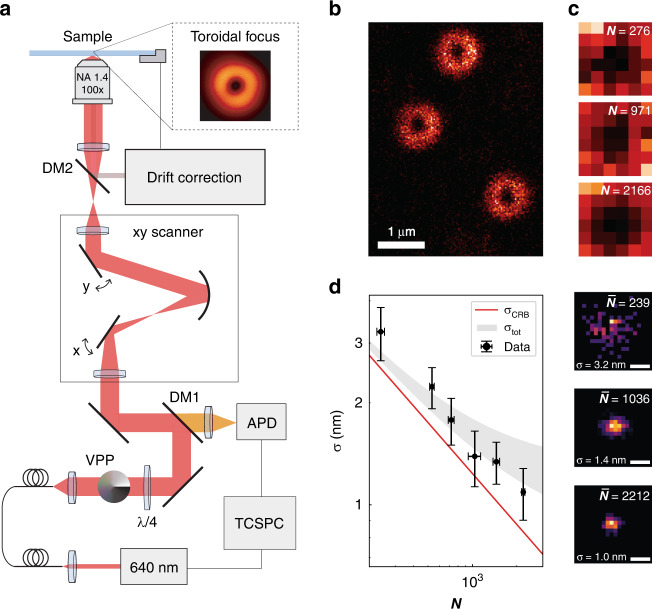
Experimental implementation in a confocal microscope. **a** Schematic representation of the optical setup used to perform RASTMIN. The setup is based on a standard home-built confocal microscope with an optical scanner. A vortex-phase plate in the excitation path and a drift correction system were added. The inset shows an image of the experimentally obtained toroidal focus. **b** Confocal image overview of the sample containing individual DNA origami with one fluorescent molecule each. **c** Exemplary RASTMIN frames obtained at different laser powers (3, 12, and 24 µW). The RASTMIN parameters were *K* = 6 × 6, *L* = 100 nm, frame time was 130 ms, and *SBR* was 4. **d** Localization precision achieved in RASTMIN measurements on single molecules (ATTO647N) with different photon counts. For each condition (*N*), three independent measurements were carried out, each one consisting of 100 localizations. For comparison, the theoretical *σ*_CRB_(*N*) (solid line) and the predicted localization uncertainty for the experiments ($\sigma _{\rm{TOT}} = \sqrt {\sigma _{\rm{DC}}^2 + \sigma _{\rm{CRB}}^2}$, gray shaded area) are shown for *σ*_DC_ = 0.8–1.3 nm. The theoretical *σ*_CRB_(*N*) was calculated in the central 10-nm part of the FOV. On the right, exemplary 2D histograms of localizations with average photon counts of $\bar {N} = 239,1036,$ and 2212. The corresponding localization uncertainties ($\sigma = 3.2,1.4,$ and $1.0$ nm) are also displayed. Scale bar in **d**: 5 nm

RASTMIN can be implemented in any fluorescence scanning microscope, e.g. confocal, capable of detecting single molecules, especially if equipped with a single-photon counting detector. Only two modifications are needed as shown schematically in Fig. 2a (see Methods and Supplementary Section 3 for further details.). First, a vortex-phase plate and polarization optics were included in the excitation path in order to generate a toroidal focus (Fig. 2a inset), as it is commonly done in the depletion beam of STED nanoscopy. Second, an active drift correction was used to maintain the lateral and axial position of the sample relative to the optical system (Supplementary Section 4). Typically, the position of the sample could be stabilized laterally within 0.8–1.3 nm (standard deviation, $\sigma _{\rm{DC}}$), and axially within 1.5–2.0 nm.

The experimental performance of RASTMIN was evaluated by localizing single fluorescent molecules (ATTO647N) attached to DNA origami structures. A detailed description of the DNA origami design can be found in Supplementary Section 5, while the sample preparation protocol is described in the Methods section. The single-molecule measurements started by scanning the excitation beam over the region of interest in the sample using low power (e.g. 5 µW at the back-focal plane of the objective) to pre-locate the individual molecules. An example image of such a scan is shown in Fig. 2b. Next, the RASTMIN measurement was performed by raster-scanning a sub-diffraction area over the target molecule, generating a so-called RASTMIN *frame*. For each single-molecule, 100 frames were acquired, and the localization precision was evaluated as the standard deviation of the 100 localizations. A detailed description of the procedure followed to estimate the localization precision can be found in the Methods section.

In order to evaluate the localization precision of RASTMIN at different photon counts, $\sigma (N)$, we carried out measurements varying the laser power and keeping the frame rate fixed (130 ms per frame). Figure 2c shows three exemplary RASTMIN frames over different single ATTO647N molecules, with laser powers of 3, 12, and 24 µW, leading to 276, 971 and 2166 photons counts per frame, respectively. A comparison between the experimental $\sigma (N)$ and the theoretical $\sigma _{\rm{CRB}}(N)$ is depicted in Fig. 2d. In addition, the predicted localization uncertainty for the experiments (gray shaded area) considering the drift correction precision ($\sigma _{\rm{TOT}} = \sqrt {\sigma _{\rm{DC}}^2 + \sigma _{\rm{CRB}}^2}$, *σ*_DC_ = 0.8–1.3 nm) is also shown. RASTMIN performs as predicted, delivering localization precisions in the 1–2 nm range for moderate photon counts (*N* = 700–2000) at *SBR* = 4.

### Fluorescence nanoscopy

**Fig. 3:**
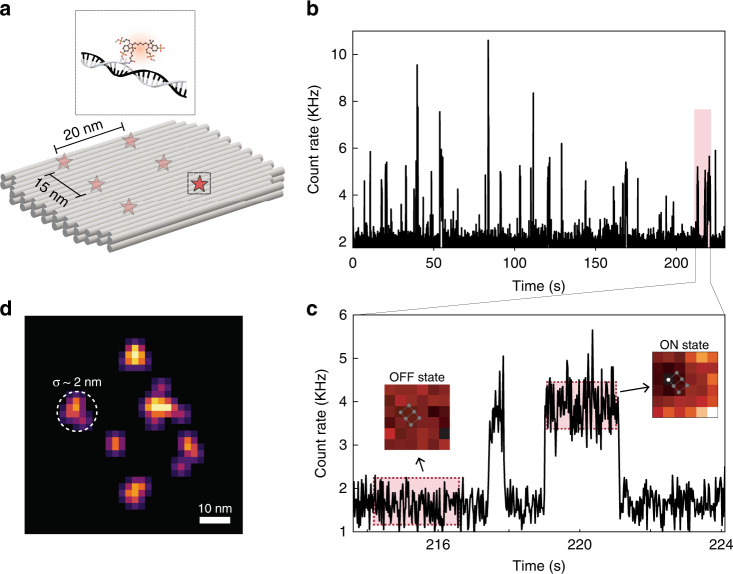
RASTMIN nanoscopy. **a** Schematic of the DNA origami used to place six fluorophores in a rectangular arrangement. **b** Fluorescence intensity time trace of representative 225 s of a RASTMIN measurement (frame time 20 ms) where single-molecule blinking events are detected. **c** Zoom-in of 10 s of the trace (highlighted region in **b**, 214–224 s) where two single-molecule emission events are observed. The inset images are the single-molecule 6 × 6 RASTMIN average frame corresponding to the periods highlighted in the time trace. During the single-molecule emission, a displaced image of a paraboloid is observed. **d** Reconstructed image of the DNA origami from the localizations obtained by RASTMIN, where a localization precision of $\sigma = 2$ nm can be observed ($\bar {N}\sim 1900$)

By combining RASTMIN with single-molecule blinking it is possible to obtain super-resolved images with nanometric resolution. To demonstrate this, a DNA origami was designed and fabricated to hold six Alexa Fluor 647 molecules organized in a regular pattern with inter-molecular distances of 15 and 20 nm, as schematically shown in Fig. 3a (more details of the DNA origami structure can be found in Supplementary Section 5).

The imaging measurements involve a multitude of blinking molecules within the sub-diffraction raster scanning area, and thus the data must be analysed to identify the single-molecule emission events. Figure 3b shows the integrated intensity (the sum of photon counts of the *K* pixels) of RASTMIN frames obtained subsequently over a DNA origami. Each RASTMIN frame ($L = 100\;{{{\mathrm{nm}}}}$ and $K = 6 \times 6$) was recorded in 20 ms and the total time of the measurement was 6 minutes. A 10 s fragment of the intensity transient is shown in Fig. 3c, where two single-molecule emission events can be observed. The emission events were discerned from background with a suitable intensity threshold as explained in Supplementary Section 6.

Before computing the molecular position, the detected emission events were processed as follows. First, events corresponding to more than one molecule were discarded based on their intensity variance, as described in Supplementary Section 6. For each single-molecule emission event, the initial and the final RASTMIN frames were discarded as they could correspond to an incomplete measurement and would therefore lead to an incorrect localization (i.e. only events lasting more than three frames were analysed). Then, all valid frames of each single-molecule emission event were summed and used to obtain a single-molecule localization. Finally, the complete set of RASTMIN localizations is rendered to reconstruct a super-resolution image as displayed in Fig. 3d. Valid RASTMIN measurements may be further filtered according to their total photon count to guarantee a minimum localization precision. In the example of Fig. 3d, localizations with $N > 450$ were used. The whole set of localizations had an average $\bar N\sim 1900$, hence yielding a theoretical expected average precision of ~1.5 nm. The super-resolved image retrieves the rectangular arrangement of fluorophores with 3×2 sites of the DNA origami with inter-molecular distances in agreement to the design. The standard deviation of the distributions of the localizations corresponding to single sites can be used as an estimate of the experimental localization precision, which is of ~2 nm.

## Discussion

RASTMIN can localize single fluorescent molecules with a precision of ~1–2 nm with photon counts *N* = 500–2000, confirming the theoretical prediction^19^. Combined with single-fluorophore blinking, RASTMIN enables super-resolution imaging with nanometric resolution, as demonstrated on DNA origami nanostructures with fixed fluorescent molecules.

A distinctive advantage of RASTMIN in comparison to other methods that can localize single molecules with similar precision is that it can be implemented on any laser-scanning microscope (e.g. confocal or multiphoton^36^), provided it has single-molecule sensitivity, with two simple modifications. First, the excitation path must be modified to produce a focus with an intensity minimum. This was done with a vortex-phase plate and suitable polarization optics as it is usually done in STED nanoscopy. Naturally, other approaches could be used such as spatial light modulators or micro-mirror arrays. Second, any method aiming to localize single molecules with nanometer precision requires the sample position to be monitored and/or stabilized to ~1 nm during the measurement time^39^. We applied a compact and effective active stabilization system that delivers a lateral stabilization precision of 0.8–1.3 nm, and of 1.5–2.0 nm for the axial direction.

RASTMIN can achieve localization precisions as high as MINFLUX with a significantly simpler setup. For example, both original^26^ and commercial^38^ implementations of MINFLUX use (i) a specialized scanning routine involving electro-optical deflectors to fine position the excitation beam coupled to a piezoelectric^26^ or galvanometric^38^ beam scanner for scanning larger sample areas, (ii) FPGA-based real-time specialized electronics to control the measurements, and (iii) closed-source software to both control the hardware and to analyze the data. While p-MINFLUX^28^ considerably simplifies the 1-nm precision measurements, we note that the experimental setup, data acquisition and analysis require expertise in synchronizing laser pulse sequences and time-correlated single-photon counting.

In contrast, RASTMIN needs only a standard raster scanning system already available in any scanning microscope and a standard DAC acquisition board and routines. Furthermore, the data acquisition is essentially the same as in any scanning microscope and the data analysis software required for the localization estimation is already available and open-source^19^. Since it is based on well-known and established technology, RASTMIN has the potential to be combined with other approaches and thus expand the applications of single-molecule localization with nanometre precision. In particular we foresee that RASTMIN could be very well-suited for a highly parallelized implementation using multiple foci^40^, in which an array of intensity light minima is scanned and multiple blinking emitters are detected simultaneously using a scientific camera or future arrays of avalanche photodiodes^41^.

Analogously to MINFLUX, RASTMIN could also be implemented in an iterative way^38^ to reduce the *L* and make the localization increasingly photon efficient, however at the cost of increasing the technical complexity. RASTMIN can also be expanded to three-dimensional (3D) single-molecule localization in different ways. For example, a 3D zero could be used, as it is used for 3D MINFLUX^30^. In analogy to 3D MINFLUX, which obtains the axial position from just two measurements, RASTMIN could retrieve axial localization based on two RASTMIN acquisitions in axially displaced planes. Alternatively, RASTMIN could be combined with SIMPLER^42^ provided a doughnut-shaped focus can be crafted in total internal reflection^43^. Since RASTMIN is fully compatible with lifetime measurements (see Supplementary Section 7), it could be directly combined with metal-induced or graphene energy transfer^44–47^.

In summary, since it is easily implementable in existing laser-scanning microscopes, we believe RASTMIN has a strong potential to be quickly adopted to visualize various material and biological systems, making super-resolution at 1-nm scale and below available to a much broader community, and thus applicable to a wider range of scientific questions.

## Materials and methods

### Optical setup

The RASTMIN setup consists essentially of a custom-made laser-scanning confocal microscope complemented with an *xyz* stabilization system. A 640 nm pulsed laser (200 ps pulse width at 40 MHz repetition rate) provided fluorescence excitation. Half-wave and quarter-wave plates were added to have circular polarization and a 0–2π vortex-phase plate was included in the path to generate a doughnut-shaped focus after the objective (Leica 100×/1.4 NA oil-immersion). Two galvanometric mirrors (Cambridge Technology, 6215H) allowed for rapid beam scanning over the sample area. The sample was mounted on an *xyz* piezo nanopositioning stage that performed the active *xyz* drift correction (with a custom-made software that monitors and corrects the sample position). The fluorescence emission was detected with an avalanche photodiode and lifetime measurements were performed using a time-correlated single-photon counting unit. A more detailed description of the optical setup and the stabilization system can be found in Sections 3 and 4 of the Supplementary Information, respectively.

### DNA origami design

The DNA origami structures used for super-resolution measurements (Fig. 3, Design 1) and to measure the RASTMIN localization precision (Fig. 2b–d, Design 2) consisted of a rectangular 2LS (2-layer sheets) structure with dimensions of 60 nm × 40 nm × 5 nm. The 2LS DNA origami was designed using CaDNAno^48^ based on a previously reported structure^49^. Design 1 was folded in the presence of six different staples modified with Alexa Fluor 647 dyes, while Design 2 was folded in the presence of a staple modified with ATTO 647 N. Further information of the DNA origami design, including the details of the modified staples of both structures, can be found in Supplementary Section 5.

### DNA origami folding

Unmodified DNA sequences were purchased from Eurofins Genomics. In short, a scaffold consisting of a vector derived from the single-stranded M13-bacteriophage genome (M13mp18, 7249 bases) and staples (100 nM, ca. 32 nts) were mixed in a 1× TAE-12 buffer (40 mM Tris, 10 mM Acetate, 1 mM EDTA, pH 8, 12 mM MgCl_2_). The solution was heated to 75 °C and ramped down to 25 °C at a rate of 1 °C every 20 min. The folded DNA origami structures were purified from excess staples strands by gel electrophoresis using a 0.8% agarose gel in TAE-12 buffer for 2.5 h at 4 V/cm. The appropriate band containing the targeted DNA template was cut out and squeezed using coverslips wrapped in parafilm. Purified DNA origami was stored at 1 nM concentration in TAE-12 buffer at −20 °C.

### DNA origami sample preparation

1.5 thickness glass-bottomed chamber slides (Lab-Tek II, Thermo Fisher Scientific) were treated with 1 M KOH for 10 min, washed (3×) with PBS, functionalized with BSA-biotin (1 mg/mL, 10 min incubation), washed (3×) with PBS and incubated for 20 min with Neutravidin (1 mg/mL). The chambers were then washed with Milli-Q water (3×) and incubated for 10 min with a solution containing 100 nm spherical gold nanoparticles in Milli-Q water. The gold nanoparticles were synthesized as described in ref. ^50^. Briefly, ∼10 nm gold seeds were prepared by heating 2.2 mM sodium citrate aqueous solution and, after boiling, adding 25 mM HAuCl_4_ solution. After 30 min, the reaction was finished. This process was repeated twice. The resulting solution was then used as a seed solution, and the process was repeated three times until the desired final size (determined by UV-visible spectroscopy and FE-SEM) was achieved. After adding the nanoparticles, the chambers were washed (5×) with TAE-12 buffer and incubated for 30 min with DNA origami samples in TAE-12 buffer (DNA origami concentration ~ 0.1 nM). Next, chambers were washed (5×) with TAE-12 and filled with imaging buffer. For measurements from Fig. 2 the imaging buffer consisted of a mixture of buffers A (90%) and B (10%), being A: 1× TAE, 12 mM MgCl_2_, 2 mM Trolox/Troloxquinone, 1 % (w/v) D-(+)-Glycose; and B: 1 mg/mL glucose oxidase, 0.4 % (v/v) catalase (50 μg/mL), 30% glycerol, 12.5 mM KCl in 50 mM TRIS. For the nanoscopy experiments (Fig. 3), the imaging buffer contained 50 mM TRIS pH 8, 10 mM NaCl, 12 nM MgCl_2_, 10% w/v D-glucose, 10 mM mercaptoethylamine, 1 mg/mL glucose oxidase and 40-μg/mL catalase.

### Fluorescent beads sample preparation

1.5 thickness glass-bottomed chamber slides (Lab-Tek II, Thermo Fisher Scientific) were treated with 1 M KOH for 10 min, washed (3×) with Milli-Q water and functionalized with a positively charged layer of Polydiallyldimethylammonium chloride (PDDA) Mw = 400,000−500,000 (Sigma-Aldrich), by incubating for 15 min a solution of PDDA (1 mg/mL in 0.5 M NaCl). Next, the chamber was rinsed with 3x Milli-Q water and incubated for 15 min with 40 nm Dark Red FluoSpheres Fluorescent Microspheres (Thermo Fisher Scientific). The beads solution was prepared directly by diluting the stock solution in Milli-Q water (5:10^6^ dilution) and was sonicated for 10 min just before being added to the chamber. After this incubation, the chamber was washed (3×) with Milli-Q water and treated for 10 min with a solution containing 100 nm spherical gold nanoparticles^50^. Finally, the sample was washed (3×) with Milli-Q water.

### Experimental determination of RASTMIN localization precision

The measurements performed to study the RASTMIN localization precision at different photon counts ($\sigma (N)$, Fig. 2b–d) were carried out using different laser powers, ranging from 3 to 24 μW at the back-focal plane of the objective, $L = 100\;{{{\mathrm{nm}}}}$, $K = 6 \times 6$ and a fixed frame duration of 130 ms. For each condition, three independent measurements consisting of 100 frames were performed. Each of these frames were individually analyzed, giving rise to 100 localizations per independent experiment.

The RASTMIN localization uncertainty (*σ*) of each measurement was determined from the standard deviation of the 100 localization events. To estimate the experimental uncertainty (${{\Delta }}\sigma$) in the determination of *σ*, a 25-frame rolling window was used to analyze different *i* subsets of localization events per experiment. The standard deviation of the resulting set of $\sigma _i$ was used as an estimation of ${{\Delta }}\sigma$. Finally, the obtained uncertainty ${{\Delta }}\sigma$ for each of the three independent measurements were averaged, and these values were represented as error bars for $\sigma (N)$ in Fig. 2d.

### Fluorescence nanoscopy data analysis

An intensity threshold was defined to discern single-molecule emission (ON) events from background. After detecting the ON events, only those lasting three or more frames were considered valid for further analysis. Within each valid event, the first and last frames were excluded from the analysis since they could correspond to an incomplete measurement and would therefore lead to an incorrect localization. The rest of the RASTMIN frames were summed, and this summed frame was then used to perform the RASTMIN localization via maximum-likelihood estimation.

To avoid multiple-emitter events, each emission event was then filtered according to a statistical criterion assuming that the number of detected photons can be described using a Poisson distribution.

Further details about data analysis can be found in Section 6 of the Supplementary Information.

## Supplementary information

Supporting Information
