# Correction: An alternative to MINFLUX that enables nanometer resolution in a confocal microscope

**DOI:** 10.1038/s41377-022-00942-1

**Published:** 2022-08-10

**Authors:** Luciano A. Masullo, Alan M. Szalai, Lucía F. Lopez, Mauricio Pilo-Pais, Guillermo P. Acuna, Fernando D. Stefani

**Affiliations:** 1 Centro de Investigaciones en Bionanociencias (CIBION), Consejo Nacional de Investigaciones Científicas y Técnicas (CONICET), Godoy Cruz 2390, C1425FQD Ciudad Autónoma de Buenos Aires, Buenos Aires, Argentina; 2 Departamento de Física, Facultad de Ciencias Exactas y Naturales, Universidad de Buenos Aires, Güiraldes 2620, C1428EHA Ciudad Autónoma de Buenos Aires, Buenos Aires, Argentina; 3 Department of Physics, University of Fribourg, Chemin du Musée 3, Fribourg, CH-1700 Switzerland

**Keywords:** Super-resolution microscopy, Imaging and sensing

Correction to: *Light: Science & Applications*

10.1038/s41377-022-00896-4 published online 30 June 2022

After publication of this article^1^, it was brought to our attention that two of the Supplementary Information “List of potentially interested researchers” and “news article” were attached by mistake, they should be removed. The original publication has been corrected.

## References

[1] Masullo (2022). An alternative to MINFLUX that enables nanometer resolution in a confocal microscope. Light Sci. Appl..

