# Supplementary material for: An alternative to MINFLUX that enables nanometer resolution in a confocal microscope
Source: Light Sci Appl. 2022 Jun 30;11:199. doi: 10.1038/s41377-022-00896-4 (PMC9247048; doi:10.1038/s41377-022-00896-4)
Supplement: Supplementary file 1 — Supporting Information [file 41377_2022_896_MOESM1_ESM.docx]

**Supporting information**

An alternative to MINFLUX that enables nanometre resolution in a confocal microscope

Luciano A. Masullo^1,2^, Alan M. Szalai^1^, Lucía F. Lopez^1^, Mauricio Pilo-Pais^3^, Guillermo P. Acuna^3^, and Fernando D. Stefani^1,2*^

^1^Centro de Investigaciones en Bionanociencias (CIBION), Consejo Nacional de Investigaciones Científicas y Técnicas (CONICET), Godoy Cruz 2390, C1425FQD Ciudad Autónoma de Buenos Aires, Argentina

^2^Departamento de Física, Facultad de Ciencias Exactas y Naturales, Universidad de Buenos Aires, Güiraldes 2620, C1428EHA Ciudad Autónoma de Buenos Aires, Argentina

^3^Department of Physics, University of Fribourg, Chemin du Musée 3, Fribourg CH-1700, Switzerland

**Table of contents**

|  | **Page** |
| --- | --- |
| **Supplementary Section 1** |  |
| Definition of *L* to compare RASTMIN and MINFLUX | 2 |
| **Supplementary Section 2** |  |
| Dependence of the localization precision with$K$ | 3 |
| **Supplementary Section 3** |  |
| Optical Setup | 4 |
| **Supplementary Section 4** |  |
| Stabilization module | 6 |
| **Supplementary Section 5** |  |
| DNA origami design | 11 |
| **Supplementary Section 6** |  |
| Fluorescence nanoscopy data processing | 13 |
| **Supplementary Section 7** |  |
| RASTMIN combined with fluorescence lifetime imaging | 16 |
| **References** | 17 |

**Supplementary Section 1**

**Definition of *L* to compare RASTMIN and MINFLUX**

In RASTMIN it is more intuitive to define $L$ as the side of the squared area defined by the size of the $K$ pixels of the image and not the diameter of the pattern of exposures. In this way, the area of the image can be directly defined by the $L$ parameter. In MINFLUX previous work, as well as in our first theoretical study of RASTMIN, $L$ is defined as the distance between the *centres* of the exposures, namely the diameter of the exposure pattern which we will call here $D$. Thus, in order to consistently compare the different methods using previously developed code^1^, the following equation has to be used:

$$L=\frac{D}{\sqrt{2}}\frac{K}{K-1}$$

Where the relationship is derived by pure geometrical considerations, as depicted in Figure S1. For example for $L_{RASTMIN}=100$ nm, $D_{MINFLUX}=D_{RASTMIN}=118$ nm was used for comparison and for $L_{RASTMIN}=50$ nm, $D_{MINFLUX}=D_{RASTMIN}=59$ nm was used.


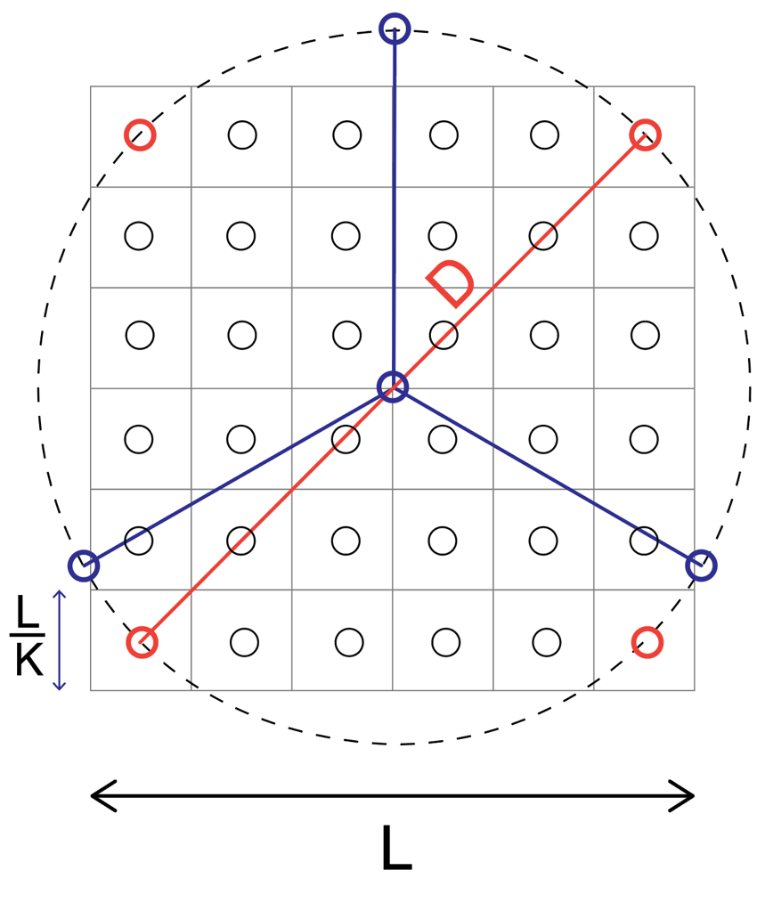


**Figure S1.** Definition of $L_{RASTMIN}$ as compared to previously defined parameters. Corner expositions of the RASTMIN pattern are depicted in red and MINFLUX excitation pattern is depicted in blue.

**Supplementary Section 2**

**Dependence of the localization precision with**$\boldsymbol{K}$

Figure S2 shows curves of $\bar{\sigma}_{CRB}$ vs $K$ for different total photon counts $N$. Also, exemplary simulated images for numbers of pixels $K=3\times3, 6\times6, 10\times10, 16\times16$ are shown. Our calculations show that as $K$ increases, the localization uncertainty decreases significantly from $K=2\times2$ up to $K=6\times6$. From then on, the localization precision does not improve substantially for higher $K$.


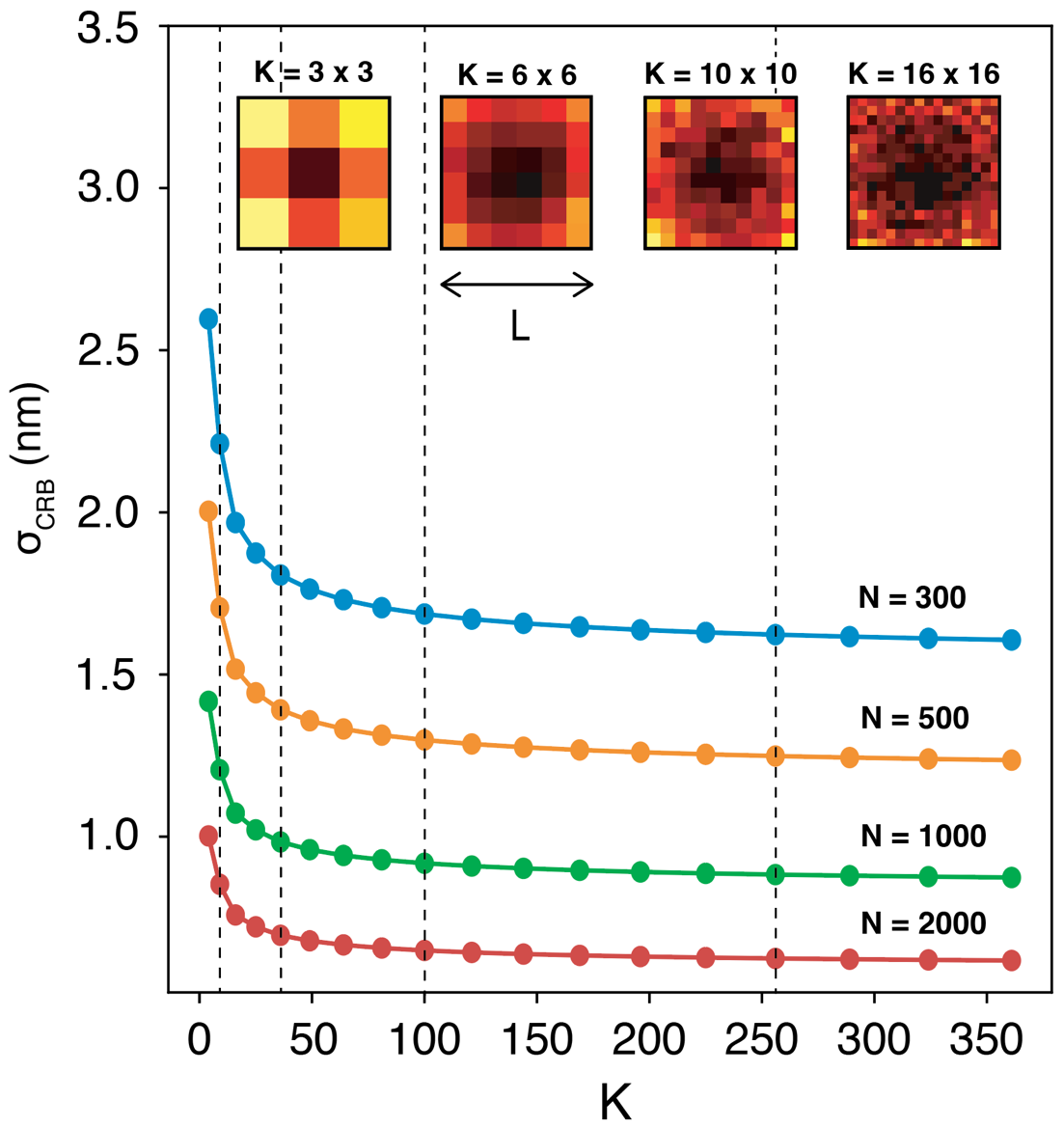


**Figure S2. Dependence of the localization precision with**$\boldsymbol{K}$**.** $\bar{\sigma}_{CRB}$ vs $K$ for different total photon counts $N$. Exemplary simulated images for numbers of pixels $K=3\times3, 6\times6, 10\times10, 16\times16$ are shown as insets and their positions in the graph are marked with black dotted lines.

**Supplementary Section 3**

**Optical Setup**

We implemented RASTMIN in a custom-made confocal microscope counting with a beam-scanner based on galvo mirrors and a piezoelectric stage to control the motion of the sample holder. The same microscope is used in our laboratory to perform STED nanoscopy. A detailed description of the setup working in its STED mode is provided in previous work^2^. Briefly, the excitation path of the home-built optical setup consists of a linearly polarized pulsed laser at 640 nm (200 ps pulse width, PicoQuant LDH-P-C-640B) operating at 40 MHz repetition, which is coupled into a polarization-maintaining single-mode fibre (Thorlabs P3-488PM-FC-5) using a fibre collimator (Schäffer + Kirchhoff 60FC-4-A7.5-01). Light exiting the fibre is collimated ($f=30 \mathrm{mm}$) in order to obtain a TEM00 excitation beam, and circular polarization is adjusted using both a broadband (400–800 nm) quarter-wave plate (Thorlabs AQWP05M-600) and a 460–680 nm half-wave plate (B.Halle Nachfl.). Then, light is directed to a 0-2π vortex-phase plate (VPP, V-633-10, Vortex Photonics), which provides the phase to generate the doughnut-shaped focus. The VPP is mounted on a stage with micrometric precision adjustable lateral position ($x,y$) and tilt ($\theta,\varphi$).

The beam is then directed to the scanner system, which consists of two lenses, two orthogonal galvanometric mirrors (horizontal, $x$, and vertical, $y$), and one concave mirror, as shown in Figure S3. The voltages required to drive the galvanometric mirrors are provided by a linear-regulated power supply (Peaktech 6060) controlled with a DAQ board (National Instruments PCIe-6353). The DAQ board is in turn interfaced via the specialized software Imspector^3^. Next, light is collimated by the tube lens of the microscope and focused into the sample with an objective with 1.4 NA (Leica HCX PL APO 100x/1.40-0.70 Oil CS). The sample is mounted on an $XYZ$ piezoelectric nanopositioning stage (Thorlabs NanoMax MAX311D/M with controller BPC303).

The emission arising from the sample passes through the same galvo-based scanning system described above, then through a long pass dichroic mirror (FF649-Di01-25x36, Semrock) and an emission band-pass filter (ET700/75m, Chroma), and is finally focused into an avalanche photodiode detector (SPCM-AQR-13, PerkinElmer Optoelectronics). The digital signal from the APD is sent to a TCSPC unit (PicoHarp 300, PicoQuant) that correlates the photon arrival signal with the laser pulses in order to obtain the fluorescence lifetime measurement. Two notch filters (ZET647NF, Chroma, and NF03-785-25, Semrock) are placed in the emission path to avoid detecting back-reflections of the excitation beam and of the IR beam used in the stabilization system (see Supplementary Section 4), respectively.

**
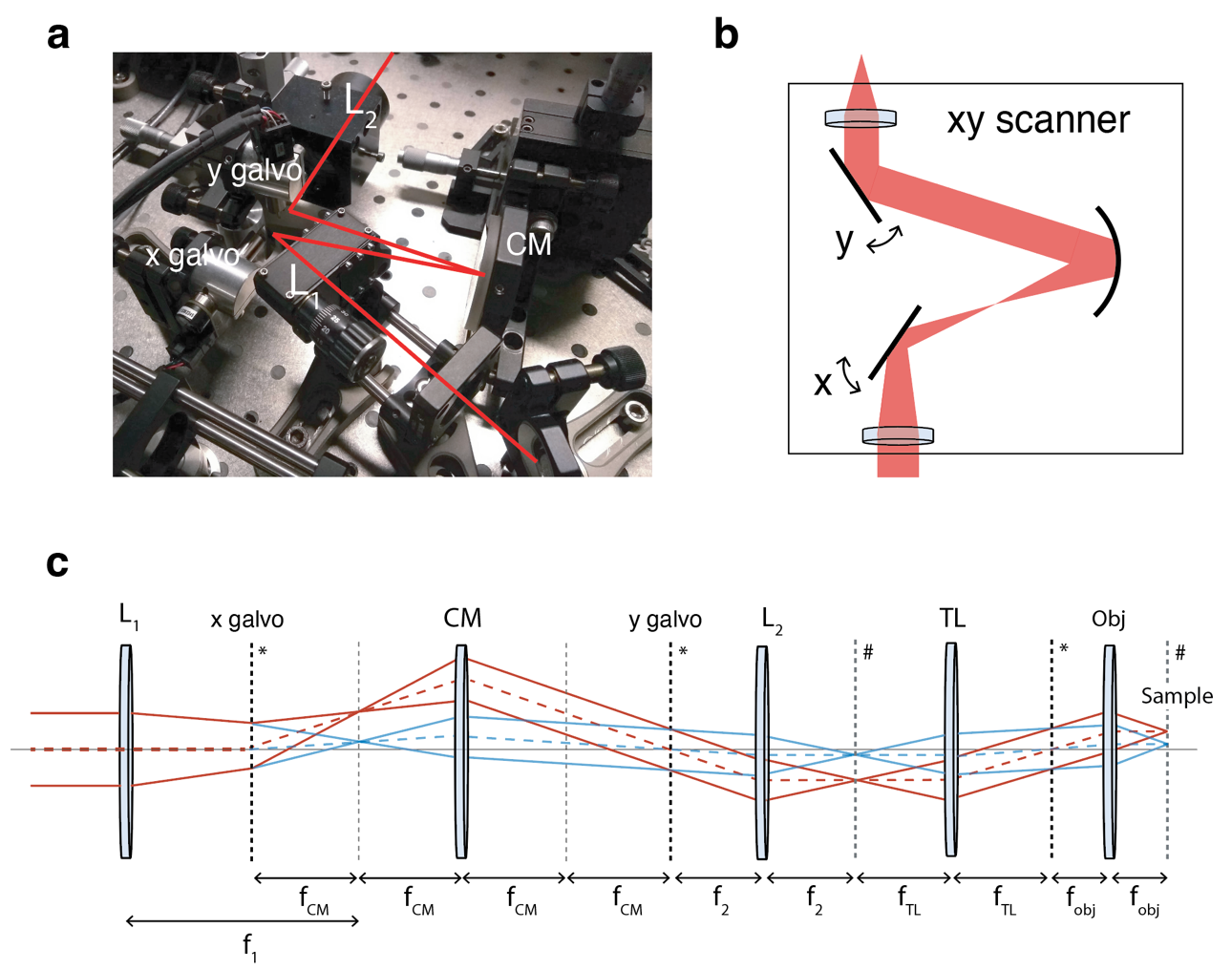
**

**Figure S3.** **Optical raster scanning. (a)** Picture of the optical elements forming the optical scanner. **(b)** 2D schematic representation. **(c)** 1D detailed representation of the positioning of each element. $L_{1}$: achromatic doublet, $f=100 \mathrm{mm}$ (Thorlabs), $L_{2}$: aberration corrected scanning lens (Leica), $f=200 \mathrm{mm}$. CM: $2’’$ concave silver mirror (Thorlabs). $x$ and $y$ galvo: galvanometric mirrors (Cambridge Technology, Inc). Conjugated planes are marked with * and #.

**Supplementary Section 4**

**Stabilization module**

In order to implement RASTMIN, we complemented the microscope with an active $xyz$ stabilization system that provides a stabilization better than $1.2 \mathrm{nm}$ in $xy$ ($\sigma_{drift x}$, $\sigma_{drift y}$) and better than $2 \mathrm{nm}$ in $z$ ($\sigma_{drift z}$). We used 100 nm gold spherical nanoparticles (AuNP) as fiducial markers, and used the light scattered by them at 775 nm as the signal to perform the active drift correction.

Figure S4 shows schematically how the stabilization system can be added into a confocal microscope by combining the excitation/fluorescence light (642 nm/670-730 nm) with IR (775 nm laser, One Five Katana HP) light through a dichroic mirror (DM, T750SPXRXT-UF1). The IR stabilization system can be divided into an excitation branch (Figure S4a) and a detection branch (Figure S4b). Both branches are divided by a 50:50 non polarizing beam-splitter ($BS_{3}$).

In the excitation branch, the IR light is coupled into a monomode fibre that provides a TEM00 beam profile and is collimated by a fibre collimator (60FC, Schäfter & Kirchhoff GmbH). It is then split into two paths by a beam splitter ($BS_{1}$). In the upper path a lens ($L_{1}$) is matched with the tube lens ($TL$) of the microscope in a $4f$ system. In this way, the beam is focused on the sample by the objective. This beam is on purpose aligned with a tilt such that the $z$ position of the sample can be inferred by the shape of the reflected beam with each $z$ plane corresponding to a different, displaced slice of the focused beam.

The lower path of the excitation is a wide-field illumination. A telescope formed by $L_{2}$ and $L_{3}$ expands the beam which is focused on the back focal plane ($BFP$) of the objective by the $TL$. The illumination is aligned such that the beam is focused at the outer part of the $BFP$, exactly as it is done in TIRF microscopy. This way the light will be reflected with an angle and, if focused again, appear as a spot off the optical axis. Both excitation paths are combined by $BS_{2}$. A third beam splitter ($BS_{3}$) is used to separate excitation light from scattered/reflected light, which is the signal that will be detected on the camera.

**
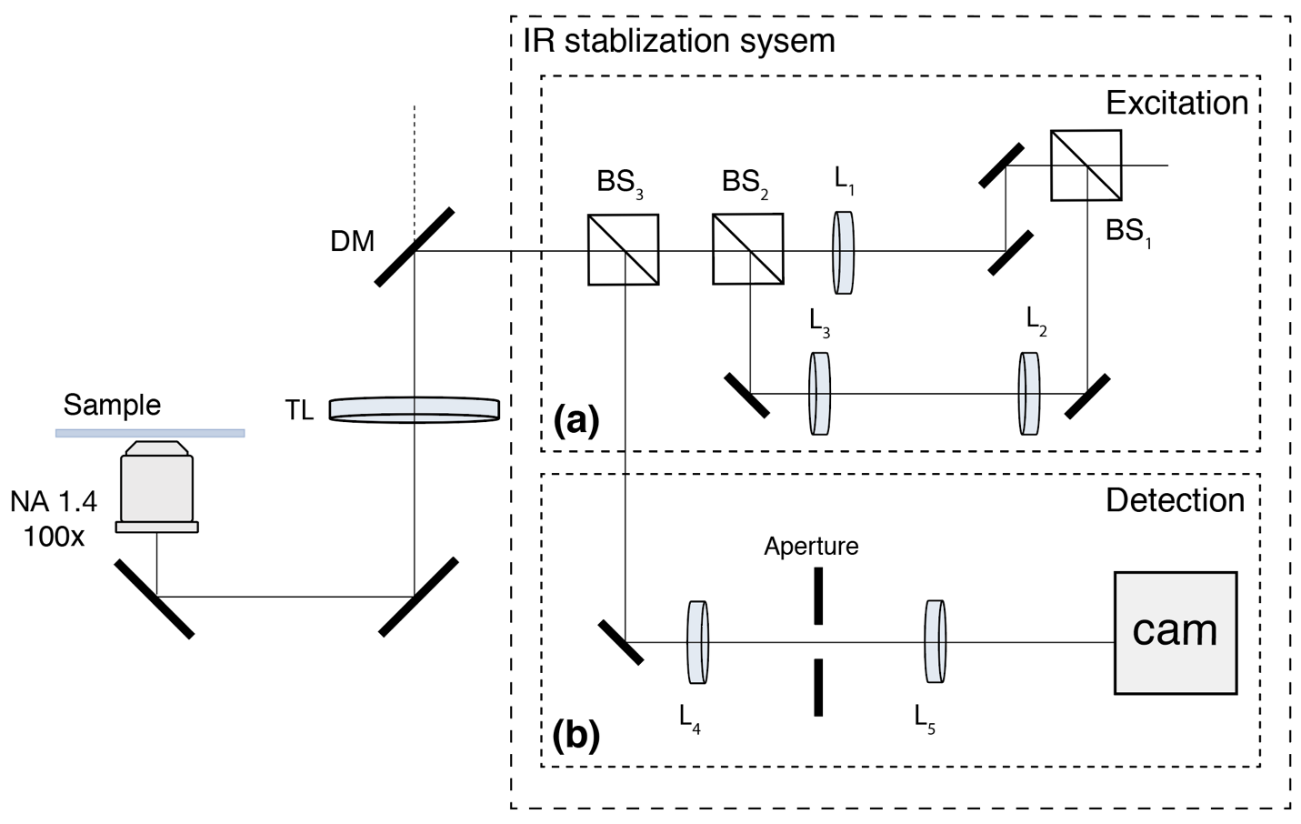
**

**Figure S4. Stabilization system – hardware. (a)** Infrared ($775 \mathrm{nm}$) illumination path. Upper part: beam for $z$ position sensing. Lower part: wide-field, TIR illumination for $xy$ fiducial localization of AuNPs. **(b)** Detection path. An aperture is placed at a conjugated plane of the BFP. The aperture blocks the reflected light from the TIR illumination, constituting an effective dark-field microscope. Both the $z$ position beam and the light scattered by the AuNPs are focused on the camera.

In the detection path, the system comprised by the objective and the $TL$ forms an intermediate image of the scattering NPs between $BS_{3}$ and $L_{4}$. On the other hand, the $4f$ system formed by the $TL$ and $L_{4}$ creates an image of the $BFP$ where an aperture is placed. This aperture can be closed to block the reflected excitation light that comes with a large angle when collimated and that is focused off-axis by $L_{4}$. The light scattered by the AuNPs arrives collimated at the aperture and has low-angle components and hence its signal is only partially blocked by the aperture. At the expense of a slight loss in image resolution (because of blocking high-angle components of the scattered light) the $SBR$ is increased dramatically as all reflected light is blocked. In this way, the IR stabilization path becomes in practice a *dark-field* imaging system (Figure S5) using standard low-cost optical components.


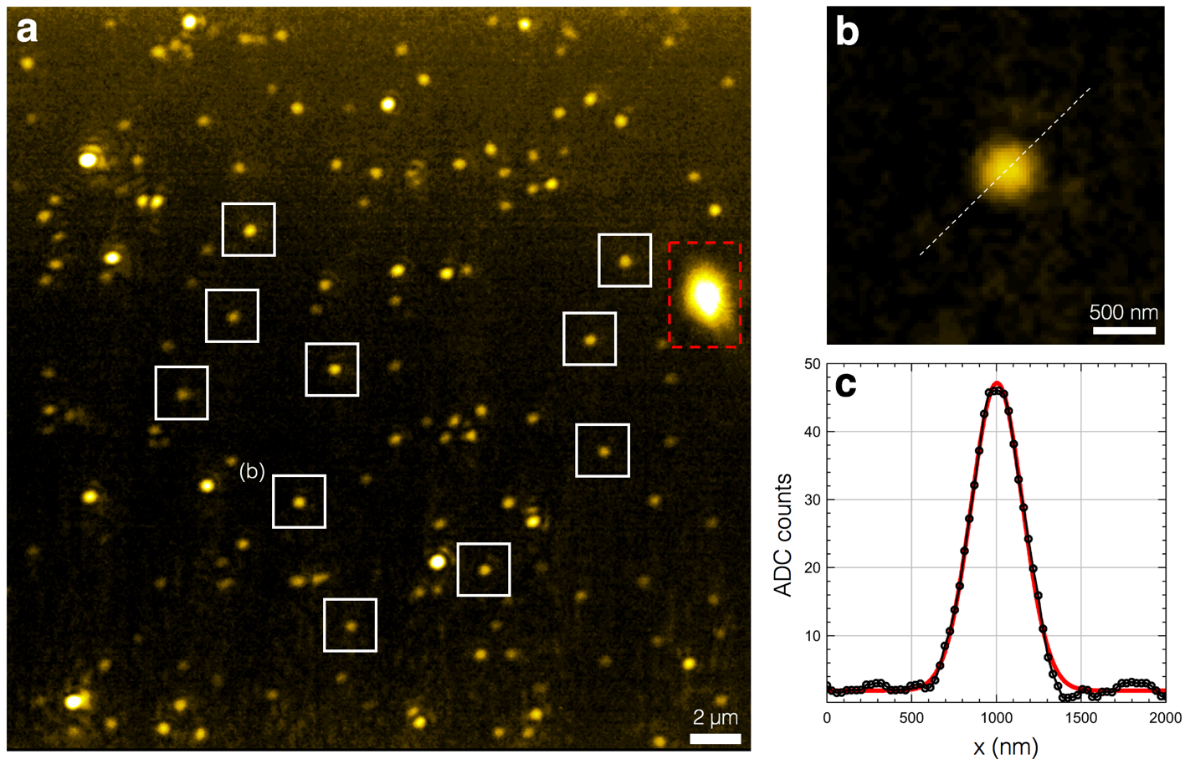


**Figure S5. (a)** The IR path effectively forms a dark-field imaging system. Single AuNPs (diameter 100 nm) are imaged with $SBR>10$. Typically, around 10 well isolated AuNPs are selected as fiducial markers (white squares). Additionally, the reflected beam to sense the axial drift is selected (red rectangle). **(b)** Close-up image of the AuNP marked in **(a). (c)** 1D intensity profile (black dots) and fit with a Gaussian function (red line).

After the aperture, another relay lens, $L_{5}$, creates an image of the AuNPs on the 8-bit sCMOS camera (ThorCam DCC1240C, Thorlabs). On the other hand, the beam used to track the $z$ position was focused on the sample and the system formed by $Objective-TL-L_{4}-L_{5}$ forms an image of the tilted focus spot on the camera.

Thus, from a single acquired image both $xy$ and $z$ position of the sample relative to the optical system can be retrieved. The lateral positions of the nanoparticles deliver the $xy$ position of the sample, and the lateral position of the reflected beam provides the information about the axial position of the sample. Then, the position of the sample is corrected actively with a piezoelectric stage.

Communication with the instruments used in the stabilization system (sCMOS camera and piezoelectric stage) is performed digitally through Python drivers. All control is integrated in a custom-made Python software that provides a graphical user-interface (GUI) to monitor the stabilization and to interact with the measurements. The architecture of the software is divided into *backend* and *frontend* classes in order to keep a clean distinction between functions that communicate with hardware (backend) and the display of the data and the commands at the GUI (frontend). The control software is open-source and can be found at <https://github.com/lumasullo/pyflux> and <https://github.com/stefani-lab/pyflux>. Figure S6 shows a screenshot of the graphical user-interface.

Images are acquired at frame rates between $10$ and $20 \mathrm{Hz}$ with 8-bit digital resolution. Single AuNPs are manually selected (Figure S6a, red squares) and their position is monitored in real-time (frame by frame) by a Gaussian fit to their intensity distribution. The IR focused beam is also selected manually (Figure S6a, yellow square) and its center of mass is tracked in real-time. Calibrations to translate lateral displacements in pixels into $(x,y)$ and $z$displacements of the sample in $\mathrm{nm}$ are obtained by performing predefined movements with the piezo-stage and registering the displacement values in pixels. By selecting *i* nanoparticles, a $(x_{i}, y_{i})$ array of estimated positions of the AuNPs is then averaged to obtain a $(\bar{x}, \bar{y})$ mean position. The latter is used as a reference of the position of the sample with respect to the optical system.

**
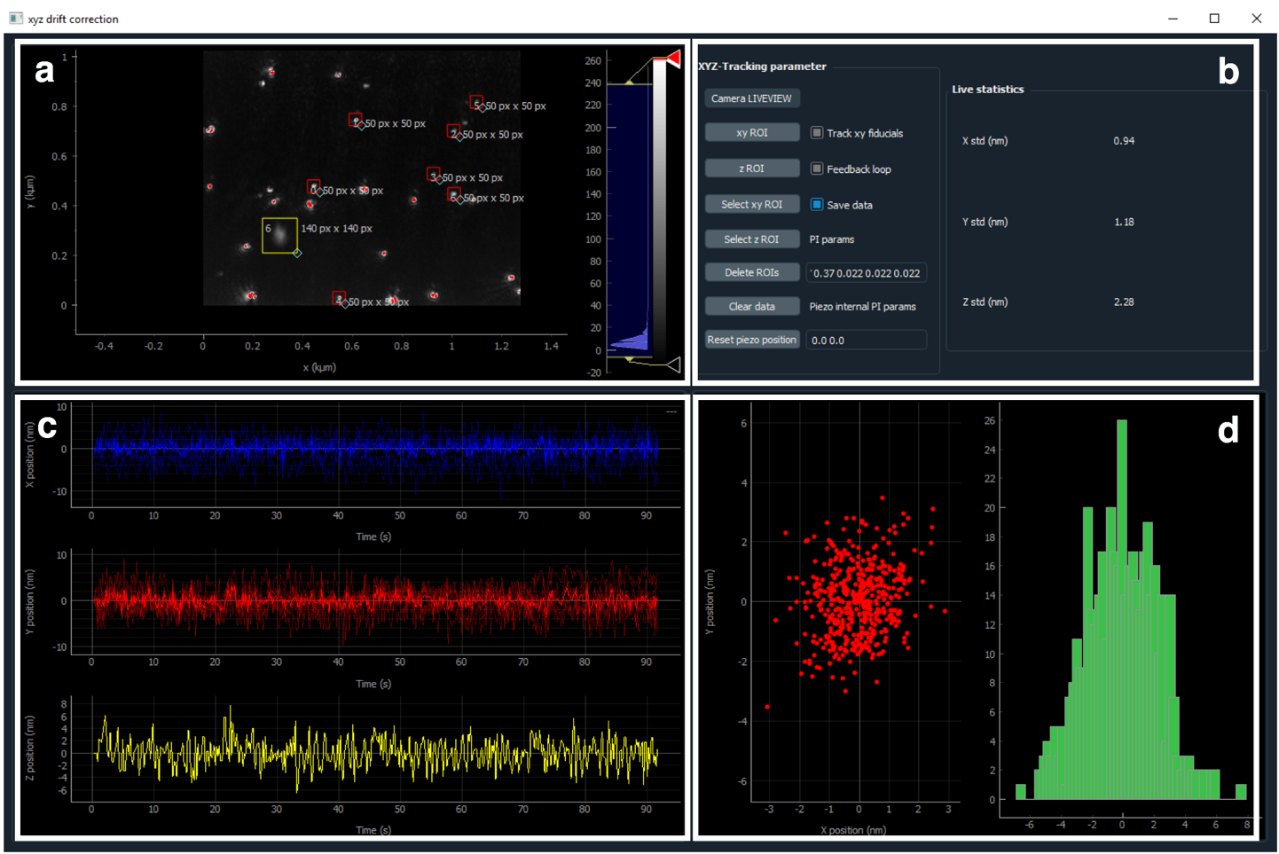
**

**Figure S6. Stabilization – instrumentation and control. (a)** Typically between 5 and 10 gold nanoparticles (AuNPs) are manually selected (red ROIs) and they are localized and tracked continuously at a frame rate of $\sim20 \mathrm{Hz}$. The reflected IR beam used to track the $z$ position of the sample is also selected (yellow ROI). By checking the signal intensity of the AuNPs, aggregates and individual particles can be distinguished. AuNPs that have a signal far from saturation are selected. **(b)** User-interface that allows to select the fiducial AuNPs, track them and control the PI feedback loop to actively stabilize the $(x, y, z)$ position of the stage. PI parameters can be selected and manual fine tune is needed to optimize performance. Statistics on the position of the tracked AuNP and the reflected IR beam are computed to have a live readout of the performance of the stabilization system. **(c)** Position signals as a function of time for $x$, $y$ and $z$. **(d)** 2D histogram for $x-y$ and 1D histogram for $z$. All graphs are updated continuously.

The performance of the stabilization system was characterized by evaluating the mean and standard deviation of the $xyz$ positions in the closed-loop mode. We recorded time series on samples with immobilized AuNPs. In all cases we chose between 5 to 10 AuNPs to stabilize the system and carefully discarded saturated spots which are probably aggregates of NPs. The exact values of the stabilization precisions may vary slightly from one experiment to another and depend on fine-tuning of the PI parameters, but the $(x, y)$ stabilization achieved is between $0.8 \mathrm{nm}$ and 1.3 nm $(\sigma_{drift})$as best- and worst-case scenario, respectively. The stabilization achieved in $z$ is slightly worse ranging between $1.5 \mathrm{nm}$ and $3 \mathrm{nm}$ $(\sigma_{drift})$. Figure S7 shows typical time traces of $x$, $y$ and $z$. The positions are normally distributed around the setpoint value and the stability is maintained over periods of time longer than 1 hour.

**
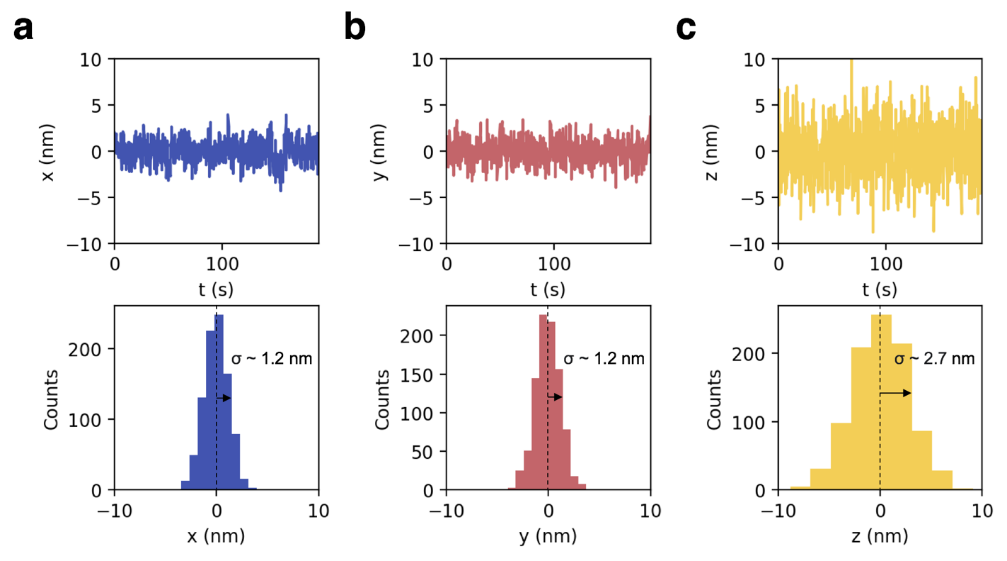
**

**Figure S7. Stability results. (a)** $x$ position of the sample vs time. **(b)** $y$ position of the sample vs time. **(c)** $z$ position of the sample vs time. All positions are relative to the setpoint (initial measured position).

**Supplementary Section 5**

**DNA origami design**

A rectangular 2LS (2 layer sheets) DNA origami with dimensions of 60 nm x 40 nm x 5 nm was designed using CaDNAno^4^ based on a previously reported design^5^. The structure integrity was verified using CanDo.^6^ The DNA template has 3 biotins on the bottom layer while the top layer is used to accommodate two distinct designs. Design 1 consists of 6x Alexa Fluor 647 fluorophores (Eurofins Genomics) modified staples. Design 2 contains a single ATTO 647N (Eurofins Genomics) modified staple (used for single molecule measurements for comparison with $\sigma_{CRB}$; Figure 2). Design 2 also contains 8x docking DNA strands extensions (Biomers) that could potentially serve as sites to perform DNA-PAINT. Figure S8 shows a screenshot of CaDNAno software, where the scaffold is marked in blue, the staples in gray, and the modified staple strands in red (6x Alexa Fluor 647 or 6x docking strands), orange (2x docking strands) and purple (ATTO 647N). The details of the modified staples are presented in Table S1.


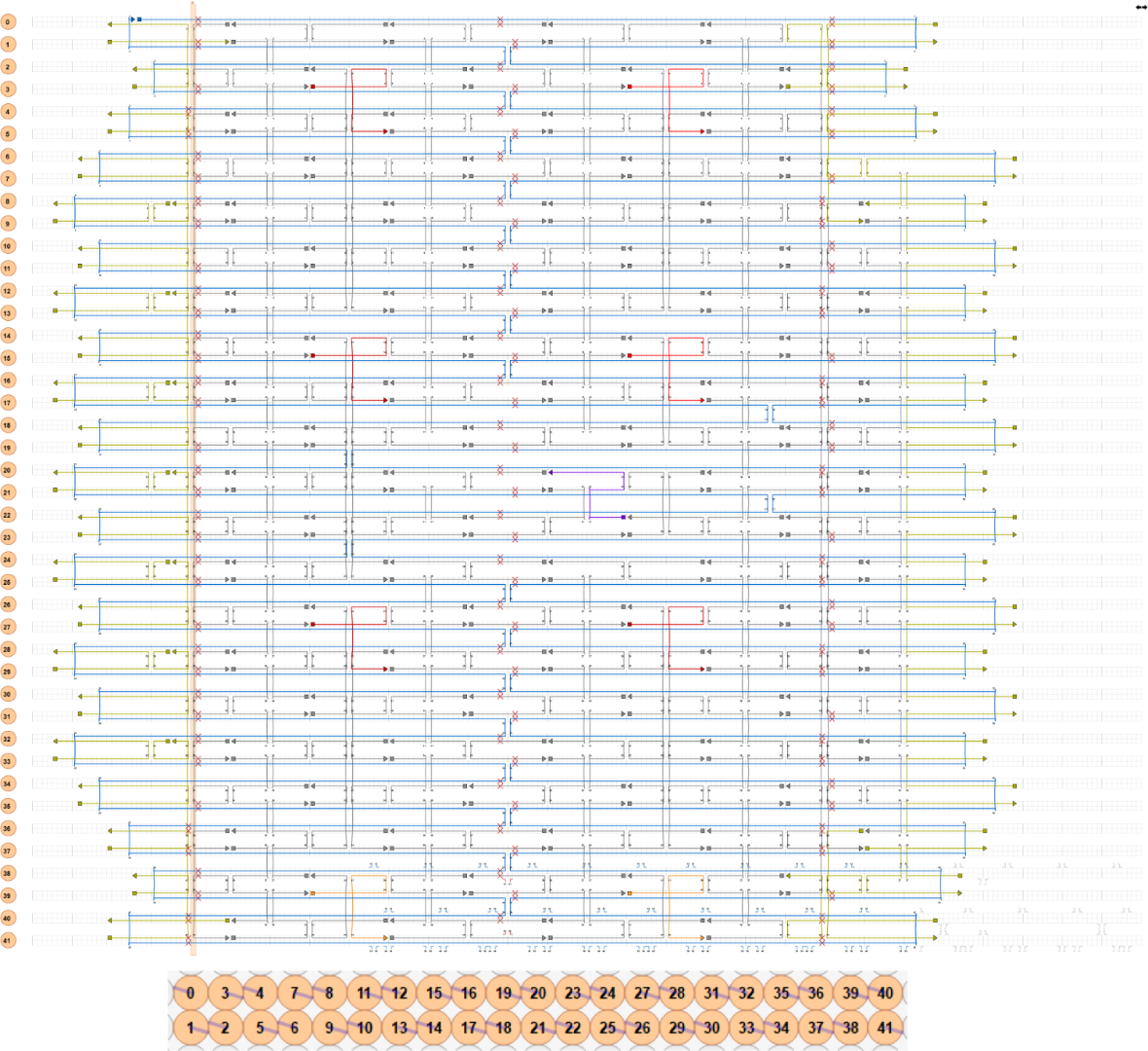


**Figure S8:** 2LS DNA origami design. Scaffold (blue), staples (gray), endcaps (green), and modified staples. Design 1 contains 6x Alexa Fluor 647 (in red). Design 2 has elongated sequences to perform DNA-PAINT (in red and orange), and staple modified with ATTO647N (purple staple). Three modified staples located at the bottom layer have a biotin modification at the 3’ end to immobilize the structure on glass substrates.

| Alexa Fluor 647  Position 1 | 5’ **[Alexa Fluor 647] TT** AGGCGCATAGGCTGGCAGTAAATTGATTATAC 3’ | **Design 1** |
| --- | --- | --- |
| Alexa Fluor 647  Position 2 | 5’ **[Alexa Fluor 647] TT** TAGCAAAATTAAGCAATATTTTAATACAAAAT 3’ |  |
| Alexa Fluor 647  Position 3 | 5’ **[Alexa Fluor 647] TT** TGCGCTCACTGCCCGCTATTGGGCTCCCCGGG 3’ |  |
| Alexa Fluor 647  Position 4 | 5’ **[Alexa Fluor 647] TT** CCAAGTACCGCACTCATCCTGAACGCTATTTT 3’ |  |
| Alexa Fluor 647  Position 5 | 5’ **[Alexa Fluor 647] TT** AAGTGTAGCGGTCACGGAGCTTGAACAGGAAC 3’ |  |
| Alexa Fluor 647  Position 6 | 5’ **[Alexa Fluor 647] TT** AATTATCATCATATTCTCGACAACAACAGTAC 3’ |  |
| Docking strand Position 1 | 5’ **TATGTAACT TT** AGGCGCATAGGCTGGCAGTAAATTGATTATAC 3’ | **Design 2** |
| Docking strand Position 2 | 5’ **TATGTAACT TT** TAGCAAAATTAAGCAATATTTTAATACAAAAT 3’ |  |
| Docking strand Position 3 | 5’ **TATGTAACT TT** TGCGCTCACTGCCCGCTATTGGGCTCCCCGGG 3’ |  |
| Docking strand Position 4 | 5’ **TATGTAACT TT** CCAAGTACCGCACTCATCCTGAACGCTATTTT 3’ |  |
| Docking strand Position 5 | 5’ **TATGTAACT TT** AAGTGTAGCGGTCACGGAGCTTGAACAGGAAC 3’ |  |
| Docking strand Position 6 | 5’ **TATGTAACT TT** GGTAATAAGTTTTAACGCTGAGACGCCAGCAT 3’ |  |
| Docking strand Position 7 | 5’ **TATGTAACT TT** AATTATCATCATATTCTCGACAACAACAGTAC 3’ |  |
| Docking strand Position 8 | 5’ **TATGTAACT TT** TAGCGACAGAATCAAGAATCACCACAGAACCG 3’ |  |
|  |  |  |
| Fixed ATTO 647N | 5’ TCAAAGCGAACCAGATGATGCAAATCCAAT **[ATTO 647N]** 3’ |  |

**Table S1:** List of modified staples for the respective DNA origami designs.

**Supplementary Section 6**

**Determination of the reference image**

The intensity profile and the center position of the excitation beam was determined after obtaining a high SNR image of individual fluorescent nanoparticles (40 nm Dark Red FluoSpheres). Tipically, a series of 16 high resolution images with a pixel size of 10 nm were acquired by scanning over a region with an isolated fluorescent bead (1.8 x 1.8 μm^2^). The images were then averaged and fitted with a 4^th^ order polynomial in two dimensions.

**Fluorescence nanoscopy data processing**

Single-molecule switching events were detected by setting an intensity threshold that allowed discerning the signal of single emitters from background. The time trace was constructed by summing the intensity values from all $K$ pixels in each frame. The intensity threshold was set to $\overline{bg}+{3\sigma}_{bg}$ as shown in Figure S9, where $\overline{bg}$ is the mean value of the background signal and $\sigma_{bg}$ is its standard deviation. Both $\overline{bg}$ and $\sigma_{bg}$ were obtained from a Gaussian fit to the histogram of intensity values as shown in the right panel of Figure S9.


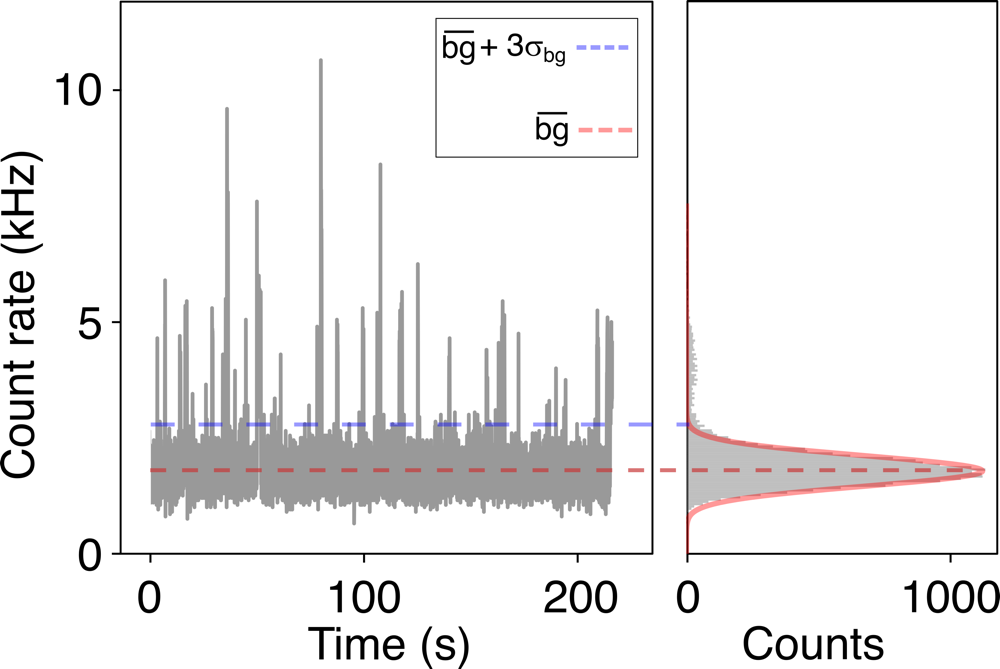


**Figure S9:** Left panel: time trace of a fluorescence nanoscopy experiment, where the mean background level (red dotted line), as well as the threshold used to discern ON events from background (purple dotted line) are depicted. Right panel: histogram of intensity values (grey) and Gaussian fit (red).

In order to filter out events where two (or more) molecules are simultaneously in their ON state, we analyzed the distribution of photons detected during each ON event and compared it with the expected value for a single molecule. The photon counts emitted by a single molecule and detected by the avalanche photodiode from our optical setup can be well described by a Poisson distribution. Thus, for each blinking event the number of detected photons in each frame, $N_{frame}$, will follow a Poisson distribution with standard deviation $\sigma_{N_{Poisson}}=\sqrt{\overline{N_{frame}}}$.

Therefore, we compared the measured $\sigma_{N_{frame}}$ with the one expected from a Poisson distribution. In the case of having more than one emitter simultaneously in the ON state, it is likely to observe at least one intensity step during the ON event, giving rise to a distribution of $N_{frame}$ that differs from a pure Poisson. In particular, $\sigma_{N_{frame}}$ is expected to be larger than $\sigma_{N_{Poisson}}$. To set a criterion to distinguish between events featuring two molecules in their ON state at the same time, we performed Monte Carlo simulations ($n_{simulations}=50000$) of Poisson-distributed events with mean value $\overline{N_{frame}}$ (in the case of the data displayed in Figure 3, $\overline{N_{frame}}=118$) and a given number of frames (for our experiments in DNA-origami measurements this value was 16). Finally, we computed the ratio $\frac{\sigma_{N_{frame}}}{\sigma_{N_{Poisson}}}$ from the simulations and set a threshold to use in our analysis pipeline to automatically detect events that significantly differ from a Poisson distribution.

In Figure S10a we show the distribution of $\frac{\sigma_{N_{frame}}}{\sigma_{N_{Poisson}}}$ obtained from these simulations and we can conclude that 99.5% of the population lies below $1.5$. Therefore, we used this value as the threshold to determine if a given ON event follows a Poisson distribution (single molecule) or not (multi-emitter). In Figure S10b an example of a two-molecule blinking event obtained from the data displayed in Figure 3 is shown. Two intensity levels can be observed. In this case, the value of $\frac{\sigma_{N_{frame}}}{\sigma_{N_{Poisson}}} =2.87$ exceeds the $1.5$ threshold and the event is discarded. On the other hand, another example obtained from our experimental data is shown in Figure S10c. In this case, $\frac{\sigma_{N_{frame}}}{\sigma_{N_{Poisson}}}=1.19<1.5$ and, therefore, the localization is considered a valid event for localization. Finally, in Figure S10d the effect of ruling out the multi-emitter events in the reconstruction of a super-resolved image is shown by comparing the reconstructed image with and without the multi-emitter filtering step.


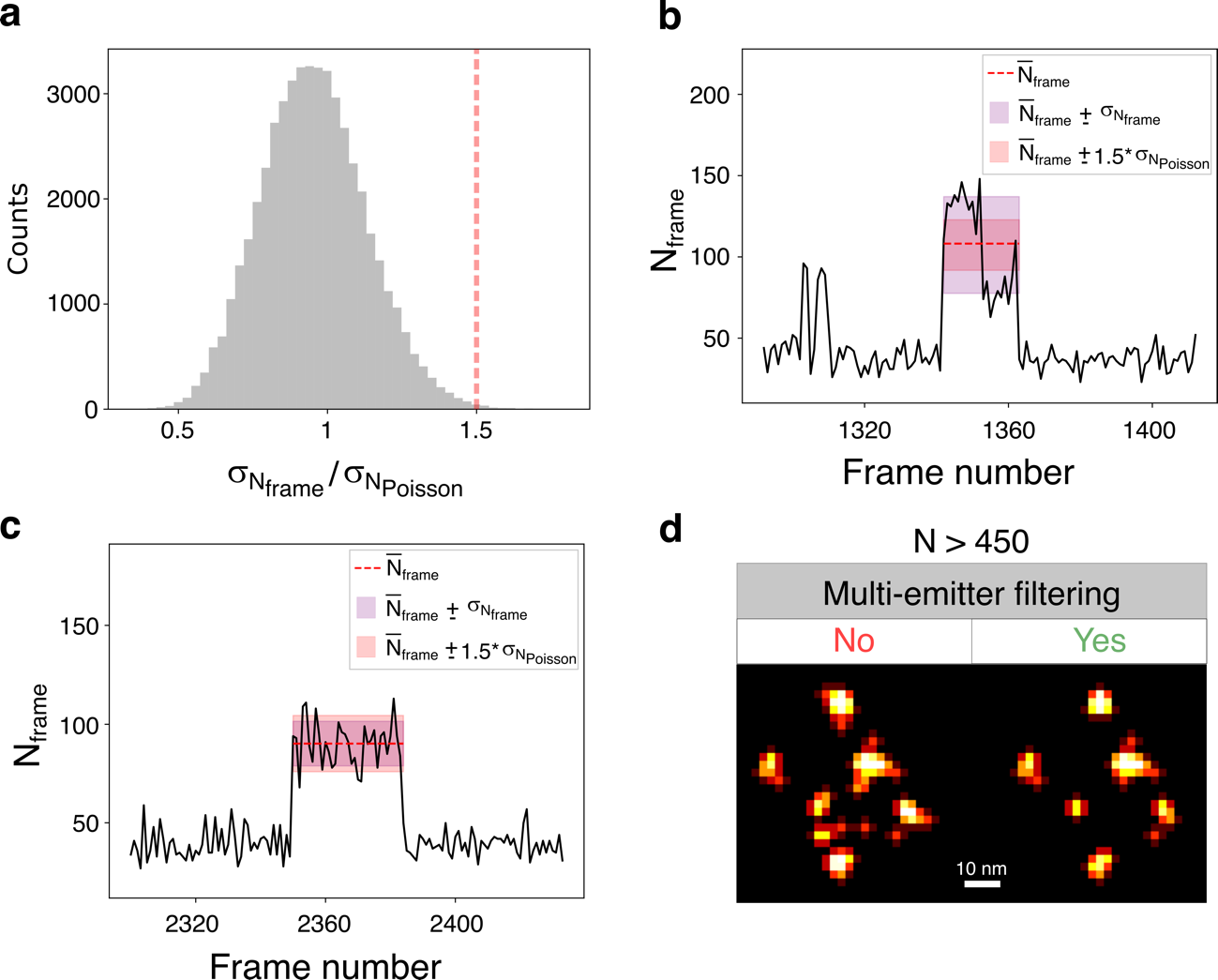


**Figure S10: (a)** Distribution of $\frac{\sigma_{N_{frame}}}{\sigma_{N_{Poisson}}}$ obtained from 50000 simulations of ON events ($\overline{N_{frame}}=118$ and 16 frames for each simulation). The threshold value of $\frac{\sigma_{N_{frame}}}{\sigma_{N_{Poisson}}}=1.5$ used to determine if ON events correspond to one or more molecules is highlighted with a dashed red line. **(b)** Example of an experimental ON event where two molecules were simultaneously in the ON state. The $\frac{\sigma_{N_{frame}}}{\sigma_{N_{Poisson}}}$ratio is higher than the one expected by a pure Poisson distribution, due to the presence of two well-differentiated intensity levels. For this reason, this localization is filtered out. **(c)** Example of an ON event where the value of $\frac{\sigma_{N_{frame}}}{\sigma_{N_{Poisson}}}$ lies within the expected range for a pure Poisson distribution. In this case, the blinking event is valid for localization. **(d)** Comparison between the reconstructed image of a DNA-origami structure when the multi-emitter filter is applied, and the image obtained when this filtering step is not performed. In both cases, only events with a total number of photons larger than 450 were considered.

**Supplementary Section 7**

**RASTMIN combined with fluorescence lifetime imaging**

To demonstrate the compatibility of RASTMIN and fluorescence lifetime imaging, we used a sample containing fluorescent nanoparticles (40-nm Dark Red FluoSpheres, Thermo Fisher Scientific). By using the piezoelectric stage, we performed controlled movements of the sample in order to place a single nanoparticle at four different positions forming a square with a side of 7 nm. Figure S11 shows the localizations obtained with RASTMIN. At each position, the sample was kept still, and 200 independent localizations were obtained with $N \sim2000$ average photon counts. As expected, RASTMIN can fully resolve emitters separated by 7 nm.

The top right panel of Figure S11 shows the fluorescence lifetime data corresponding to the localization marked with a white dotted circle in the top left panel. The lifetime information was obtained using time-correlated single-photon counting detection. So far, the possibility to perform lifetime measurements with sub-10 nm spatial resolution had only been demonstrated by pulsed-interleaved MINFLUX (p-MINFLUX)^7^. RASTMIN attains this with a slightly modified confocal setup and a simpler measurement pipeline.


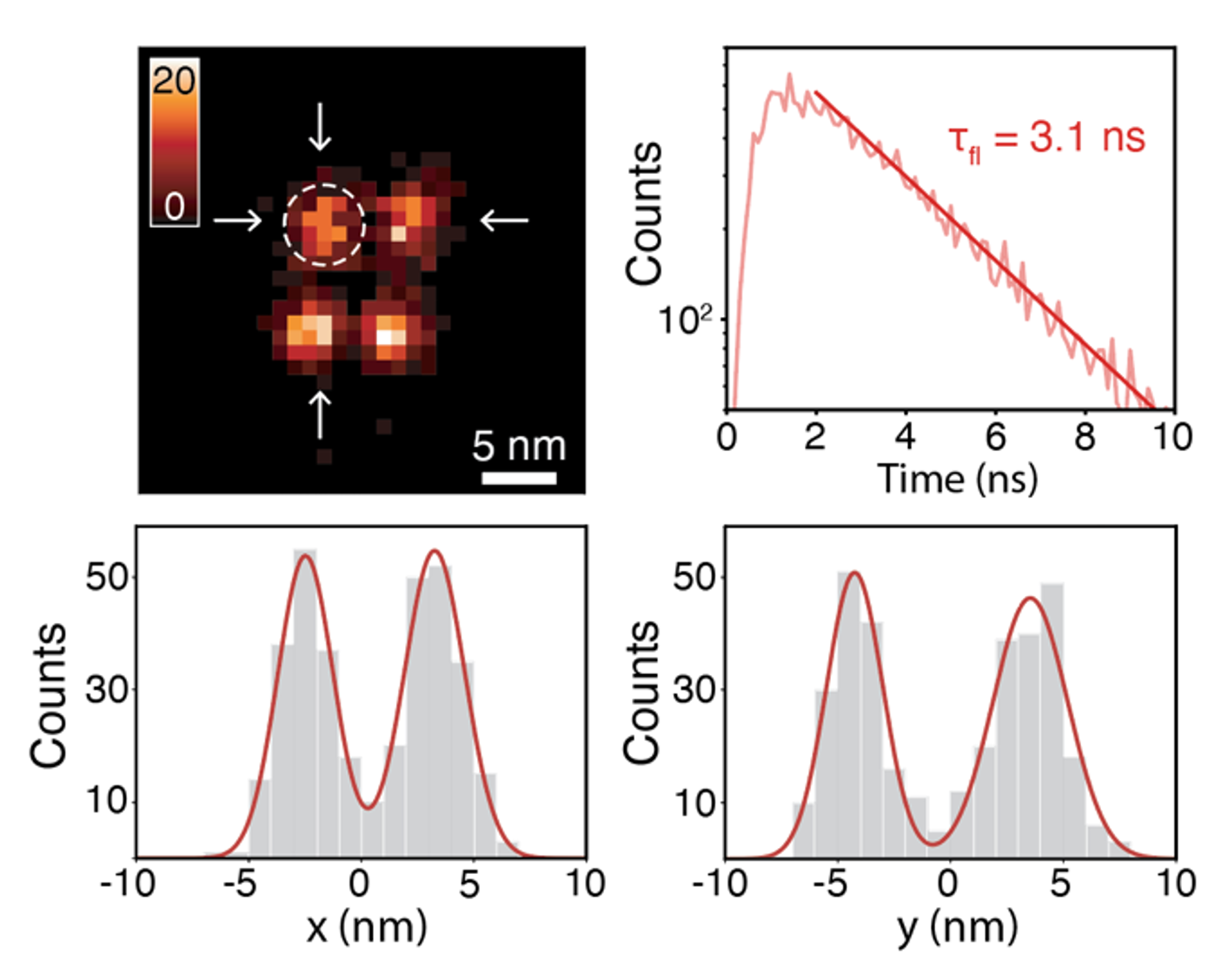


**Figure S11: Top left**: 2D histogram of the localizations obtained by placing a fluorescent nanoparticle in four positions forming a square with a side of 7 nm. **Top righ**t: fluorescence lifetime decay curve corresponding to the particle marked with a dotted white circle in the first panel. **Bottom**: 1D histograms of the regions marked with arrows in the top left panel show that distances as small as 7 nm can be fully resolved.

**References**

1. Masullo, L. A., Lopez, L. F. & Stefani, F. D. A common framework for single-molecule localization using sequential structured illumination. *Biophys. Reports* **2**, 100036 (2022).

2. Szalai, A. M. *et al.* Super-resolution Imaging of Energy Transfer by Intensity-Based STED-FRET. *Nano Lett.* **21**, 2296–2303 (2021).

3. Abberior Instruments Development Team, Imspector Image Acquisition & Analysis Software, http://www.imspector.de. (2013).

4. Douglas, S. M. *et al.* Rapid prototyping of 3D DNA-origami shapes with caDNAno. *Nucleic Acids Res.* **37**, 5001–5006 (2009).

5. Roller, E. M., Argyropoulos, C., Högele, A., Liedl, T. & Pilo-Pais, M. Plasmon-Exciton Coupling Using DNA Templates. *Nano Lett.* **16**, 5962–5966 (2016).

6. Kim, D.-N., Kilchherr, F., Dietz, H. & Bathe, M. Quantitative prediction of 3D solution shape and flexibility of nucleic acid nanostructures. *Nucleic Acids Res.* **40**, 2862–2868 (2012).

7. Masullo, L. A. *et al.* Pulsed Interleaved MINFLUX. *Nano Lett.* **21**, 840–846 (2021).
